# Oral lipoteichoic and lipoic acids improve insulin resistance and body composition in porphyria mice on a high-carbohydrate diet

**DOI:** 10.1007/s13105-025-01124-4

**Published:** 2025-09-26

**Authors:** Miriam Longo, Teresa Rubio, Araceli Lamelas, Daniel Jericó, Andrea Rodenes-Gavidia, Jordi Cervero, Juan Martínez-Blanch, Empar Chenoll, Patricia Martorell, Erika Paolini, Marica Meroni, José Ignacio Riezu-Boj, Isabel Solares, Ana Sampedro, Francesco Urigo, María Collantes, Michele Battistin, Stefano Gatti, Gemma Quincoces, Ivan Peñuelas, María Jesús Moreno-Aliaga, Matías A. Ávila, Elena Di Pierro, Daniel Ramón, Fermín I. Milagro, Paola Dongiovanni, Antonio Fontanellas

**Affiliations:** 1 Medicine and Metabolic Diseases, Fondazione IRCCS Ca’ Granda Ospedale Maggiore Policlinico, Milan, 20122 Italy; 2 ADM Research Center-Valencia, University of Valencia Science Park (Parc Científic de La Universitat de València), Paterna, 46980 Spain; 3 Hepatology: Porphyrias & Carcinogenesis Lab. Solid Tumors Program, CIMA-University of Navarra, Pamplona, 31008 Spain; 4 Center for Nutrition Research and Department of Nutrition, Food Sciences and Physiology, Faculty of Pharmacy and Nutrition, University of Navarra, Pamplona, 31008 Spain; 5 Navarra Institute for Health Research (IdiSNA), Pamplona, 31008 Spain; 6 Rare Disease Unit, Internal Medicine Department. Clinica Universidad de Navarra (CUN), Pamplona, 31008 Spain; 7 Translational Molecular Imaging Unit (UNIMTRA), University of Navarra, and Nuclear Medicine-Department, Clínica Universidad de Navarra (CUN), University of Navarra, Pamplona, 31008 Spain; 8 Center for Preclinical Research, Fondazione IRCCS Ca’ Granda Ospedale Maggiore Policlinico, Milan, 20122 Italy; 9 Department of Pathophysiology and Transplantation, Università Degli Studi Di Milano, Milan, 20122 Italy; 10 Centro de Investigación Biomédica en Red de Fisiopatología de la Obesidad y Nutrición (CIBEROBN), Instituto de Salud Carlos III, Madrid, 28029 Spain; 11 Hepatology: Metabolism, Epigenetics and Carcinogenesis group, Solid Tumors Program, CIMA-University of Navarra, Pamplona, 31008 Spain; 12 Centro de Investigación Biomédica en Red de Enfermedades Hepáticas y Digestivas (CIBERehd), Instituto de Salud Carlos III, Madrid, 28029 Spain; 13 Cancer Center Clínica Universidad de Navarra (CCUN), Pamplona, 31008 Spain; 14 Department of Animal Production and Health, Public Veterinary Health and Food Science and Technology, School of Veterinary Medicine, Universidad Cardenal Herrera-CEU, Valencia, 46115 Spain

**Keywords:** Metabolic disease, Acute intermittent porphyria mice, Hyperinsulinemia management, Oral postbiotic, Insulin-mimetic agent, Dietary supplement

## Abstract

Acute intermittent porphyria (AIP) is a genetic metabolic disorder characterized by neurovisceral attacks. Although high-carbohydrate diets or intravenous glucose administration can help alleviate incipient attacks in patients, these interventions may also promote insulin resistance and increase metabolic risk. This study explored targeted dietary interventions to manage hyperinsulinemia and to enhance glucose uptake in insulin-sensitive organs under high-carbohydrate diet. Body composition and fecal microbiota profile were also investigated in a murine model of the disease. Wild-type and AIP mice (*n* = 6/group) were supplemented with tapioca maltodextrin in drinking water for 12 weeks, alongside heat-treated *Bifidobacterium animalis* subsp. *lactis* CECT-8145 (BPL1®HT), its by-product lipoteichoic acid (LTA), or the insulin-sensitizing agent α-lipoic acid (α-LA). Liver-targeted therapies, previously assessed in AIP mice, were also included in this study. AIP mice on a high-carbohydrate diet exhibited hyperinsulinemia and tissue-specific differences in glucose uptake compared to wild-type mice. Dysbiosis, marked by reduced fecal *Dorea spp.* and *Adlercreutzia muris*, alongside higher abundance of *Escherichia coli,* was also showed. Supplementation with α-LA and LTA revealed superior ability to improve glucose tolerance test and skeletal muscle glucose uptake, reduce hyperinsulinemia, and enhance body composition by increasing lean mass relative to fat, compared to gene therapy or liver-targeted insulin administration. Notably, LTA restored fecal microbiota profiles resembling those of wild-type mice. In conclusion, supplementation with LTA from BPL1®HT and α-LA may represent promising dietary interventions to manage glucose tolerance, improve insulin sensitivity in muscle and adipose tissues, and potentially ameliorate body composition in AIP patients under a high-carbohydrate diet.

**Supplementary Information:**

The online version contains supplementary material available at 10.1007/s13105-025-01124-4.

## Introduction

Acute Intermittent Porphyria (AIP) is an inherited metabolic disorder resulting from haploinsufficiency of the *hydroxymethylbilane synthase* (*HMBS*) gene, which encodes porphobilinogen (PBG) deaminase (PBGD), the third enzyme in the heme synthesis pathway. Patients might experience acute attacks, which are characterized by intense pain primarily in the abdomen, along with gastrointestinal symptoms such as nausea, vomiting, and/or constipation. These episodes may involve dysfunction of the autonomic, peripheral, and central nervous systems. Porphyria attacks are associated with the upregulation of the first and rate-limiting enzyme of hepatic heme biosynthesis, 5-aminolaevulinic acid (ALA) synthase (ALAS), leading to the accumulation of the potential neurotoxic porphyrin precursors ALA and PBG [5, 36]. Several exogenous and endogenous factors upregulate ALAS1 expression and trigger acute attacks.

A unique nutritional aspect of AIP is that fasting induces acute attacks, while a diet with high carbohydrate intake is advised to increase insulin levels and potentially downregulate the expression of the hepatic ALAS gene [16] through the PI3-K/Akt [3, 14, 35, 37]. Although, there is currently no direct evidence in humans that this intervention reduces ALA/PBG levels or biochemical disease activity, diets high in carbohydrate (> 60% of total dietary energy), along with fruit juices, candies or intravenous glucose loading have been proposed for both preventing and managing mild acute porphyria attacks [15, 31, 35, 37]. However, high glycemic diets have detrimental metabolic effects, with the most pronounced impact seen in insulin-resistant conditions such as type 2 diabetes (T2DM) or during pregnancy. Patients with AIP also show higher incidence of hyperinsulinemia and metabolic risk reliant on high-carbohydrate intake [12, 34, 35], which may impact on body composition and the efficacy of injectable glucose-based therapies [10, 15, 17, 18, 23, 24, 35]. Currently, there are no approved therapeutic interventions available to correct insulin resistance and anomalies in carbohydrate metabolism, which are primarily observed in cases of asymptomatic or latent AIP (which constitute over 90% of carriers of the HMBS gene mutation) [35].

The beneficial effects of α-LA on glucose tolerance, as an insulin sensitizer agent, have been previously demonstrated in rodents affected by obesity and T2DM [28]. Furthermore, our earlier studies introduced the concept that α-LA may enhance glycogen catabolism, gluconeogenesis and ATP content in *PBGD*-silenced hepatocytes [25], as well as in AIP mice under a short-term regimen [25].

The dietary consumption of fermented foods and beverages is widely adopted to confer health benefits in metabolic disorders such as obesity, T2DM, and non-alcoholic fatty liver disease, primarily by influencing the composition of the gut microbiota. Several factors can influence the microbiota, including host genetics, diet, physical and psychological stress, and age [29]. A high-carbohydrate diet, as received by patients with AIP, can also induce changes in the fecal microbiota and exacerbates hyperinsulinemia and fat accumulation. Therefore, it is important to identify the microorganisms involved in phenotypical improvement. *Bifidobacterium* and *Lactobacillus* are the most widely studied microorganisms used to control body weight (BW) and to reduce hepatic fat accumulation because these strains do tolerate the heat and production processes of fortified-foods. Emerging studies have highlighted that *Bacillus coagulans* has shown anti-obesity effects and protected against the metabolic disturbances induced by a high-fat diet in mice [39]. In the mouse model of AIP, supplementation with *B. coagulans* spores improved glucose tolerance, reduced hyperinsulinemia, and increased the lean/fat tissue ratio [26], however, these effects were not evaluated under a high-carbohydrate diet.

The term postbiotic refers to a preparation containing inanimate microorganisms or bioactive compounds produced by or released during microbial activity, which confer health benefit to the host. These metabolites support intestinal microbiota balance, maintain host homeostasis, and enhance epithelial cells survival in the intestine, thereby reducing pathogen-induced inflammation [22].

In this study, we provided dietary supplements to AIP mice, which exhibit hyperinsulinemia along with increased hepatic gluconeogenesis and ketogenesis resulting from their impaired utilization of glycogen stores [10]. To mimic the high carbohydrate intake observed in patients with AIP [36], tapioca maltodextrin (TM) was added to the drinking water. Additionally, we evaluated the efficacy of liver-targeted treatments previously assessed in AIP mice, including adeno-associated vector (rAAV)-based gene therapy and an insulin-apolipoprotein AI fusion protein (Ins-ApoAI) [1]. This study aims to evaluate the potential of targeted dietary supplements to manage hyperinsulinemia and to enhance glucose uptake in insulin-sensitive organs under high-carbohydrate diet.

## Materials and methods

### Study design

**Fig. 1:**
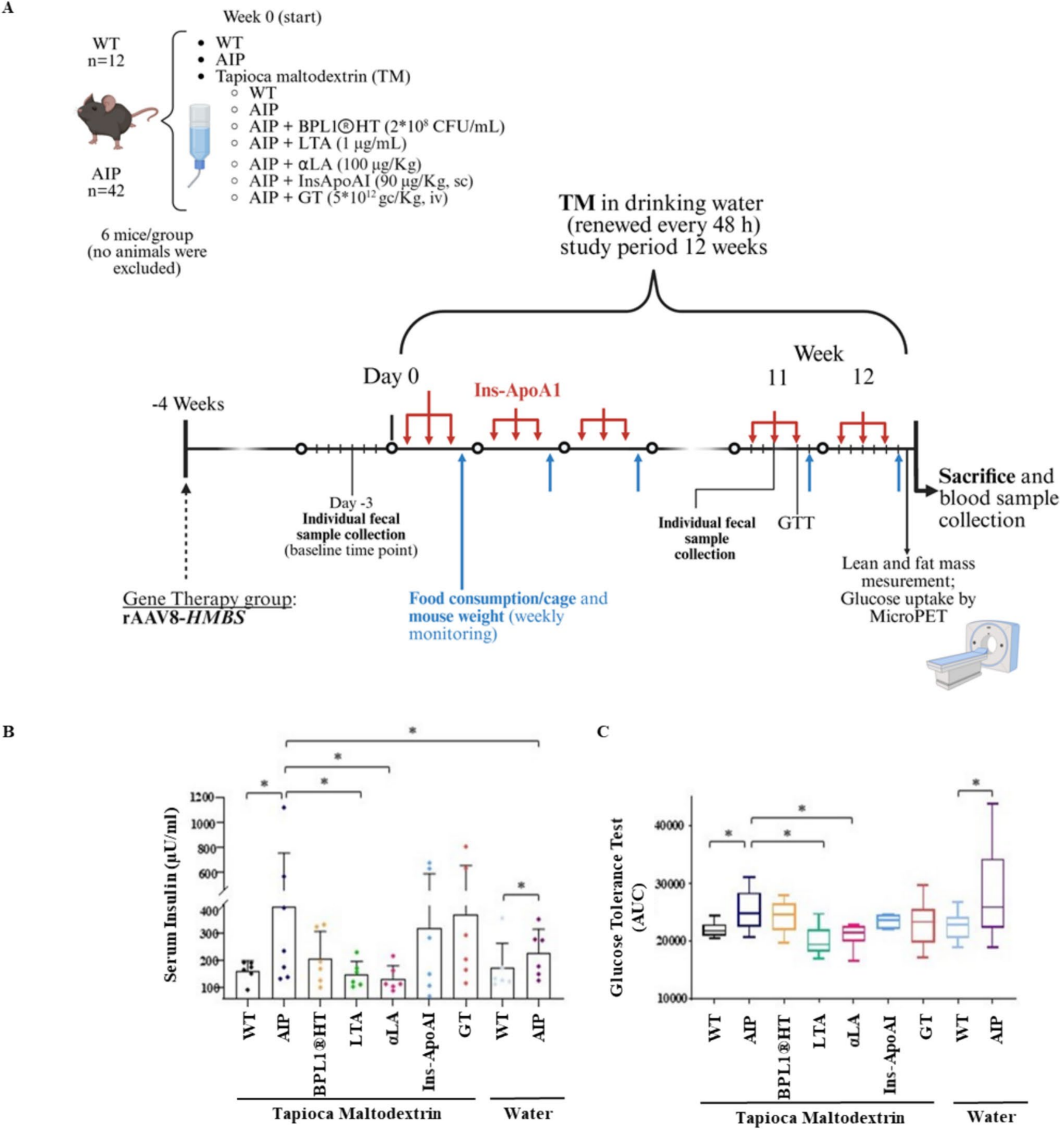
LTA and α-LA dietary interventions reduce hyperinsulinemia and improve glucose tolerance in AIP mice. **A**) Schematic timeline representing the study design and workflow. **B**) Serum insulin levels and **C**) Box plot showing the area under the curve (AUC) from glucose tolerance test (GTT), calculated between 1 and 3 h after intraperitoneal glucose administration (5 g/kg). All mice were fasted for 14 h prior to testing. Data are presented as mean ± SD (*n* = 6 per group). Statistical comparisons were performed using one-way ANOVA followed by the Bonferroni post-test. *P < 0.05 and **P < 0.01

Three-month-old female C57BL/6 wild-type (WT) and compound heterozygous C57BL/6^pbgd1(neo)Uam/pbgd2(neo)Uam^ AIP mice were randomly assigned (*n* = 6/group) to receive daily dietary supplements *ad libitum* for 12 weeks. Animals within each group were housed together in the same cage to reduce psychological stress associated with isolation. Female mice were used because porphyria is more prevalent in women [5] and to minimize the additional stress that may result from aggressive behavior among unrelated males. Supplements were diluted in water containing TM (2 mg/ml) to stabilize the substances and simulate high carbohydrate intake*.* Two AIP cohorts received either BPL1®HT (2 × 10^8^ CFU/ml), originally isolated from fresh feces of breastfed infants [8], or one of its by-products, lipoteichoic acid (LTA, Biopolis S.L., Valencia, Spain). Another AIP group received α-LA (100 mg/kg) in water, while a separate group was subcutaneously injected with the liver-targeted Ins-ApoAI (90 µg/kg) every three days to evaluate the long-term effects on a high-glycemic diet. Additionally, one AIP group received a single intravenous (i.v.) injection of 5 × 10^12^ genome copies per kilogram of rAAV5-*HMBS* vector (GT-TM) four weeks prior to the study. Control groups included untreated WT and AIP mice receiving only water or TM in water (WT-TM and AIP-TM) as negative controls. All groups were maintained on a standard diet. A schematic representation of the study design is shown in Fig. 1A. The experimental protocol was approved by the Ethics Committee of the University of Navarra (CEEA 023–22), according to the European Council guidelines.

### ***In vivo*** analysis conducted at the conclusion of the study

Analyses were conducted after 14 h of fasting. A *glucose tolerance test* (GTT, 5 g/kg, i.p.) was performed under conditions of severe hyperglycemia (blood glucose > 300 mg/dl, five minutes after the challenge, median of 420 mg/dl, IQR [389–475]). Blood glucose was measured from tail vein samples using an Accu-chek glucometer (Roche Diagnostics AVIVA, Mannheim, Germany).

One week later, *lean and fat mass* were measured using quantitative magnetic resonance through an EchoMRI analyzer (EchoMRI-100–700, Echo Medical System, Houston, TX, USA), as reported [26]. Then, *glucose uptake in some tissues* was assessed with micro-positron emission tomography (MicroPET) using the glucose analog [^18^F]Fluoro-2-deoxy-2-D-glucose ([^18^F]FDG). Imaging was performed using a Philips Mosaic tomograph (Cleveland, OH, USA), with anatomical images acquired in a U-SPECT6/CT scanner (MILabs). Mice received 9.2 ± 0.9 MBq [^18^F]FDG intravenously and were anesthetized with 2% isoflurane during a 50-min uptake period before the 15-min image acquisition. A subset underwent cold stimulation (4ºC) for 1 h before the [^18^F]FDG injection and during the uptake period. Images were reconstructed applying dead time, decay, random and scattering corrections into a 128 × 128 matrix with a 1 mm voxel size. Then, images were analyzed with PMOD software (PMOD Technologies Ltd., Adliswil, Switzerland), converted to Standardized Uptake Value ([tissue activity concentration (Bq/cm3)/injected dose (Bq)] × body weight in g). For semiquantitative analysis of the images, [^18^F]FDG uptake by brown adipose tissue (BAT) was evaluated drawing spherical volume-of-interest (radius = 2 mm) on PET images over the interscapular BAT, the brain, heart, liver and hindlimb’s muscle. A semi-automatic segmentation captured voxels > 50% of the maximum value to calculate the mean Standardized Uptake Value.

*Ex vivo* [^18^F]FDG uptake in tissues was quantified immediately after PET imaging. Animals were sacrificed, and liver, skeletal muscle (gastrocnemius), white adipose tissue (WAT) fractions including gonadal (GON), retroperitoneal (RP), mesenteric (MES) and subcutaneous (SC) and the interscapular BAT, were collected, weighed, and measured for radioactivity using a gamma counter (Hidex Automatic Gamma Counter). [^18^F]FDG uptake was expressed as % of the injected dose per gram, corrected for 18-fluorine decay.

### Serum insulin levels

Serum insulin levels were measured using a solid-phase sandwich ELISA, as previously reported [26].

### Western blot analysis

Proteins were extracted from the liver, skeletal muscle (gastrocnemius), WAT (gonadal) and BAT using RIPA buffer containing 1 mmol/L Na-orthovanadate, 200 mmol/L of phenylmethyl sulfonyl fluoride, and 0.02 μg/μL of aprotinin. Equal protein amounts (50 μg) were separated by SDS-PAGE, transferred to nitrocellulose membrane (BioRad, Hercules, CA), and incubated overnight with specific antibodies. Murine antibodies included anti-Glut2 (sc-518022, Santa Cruz Biotechnology, Heidelberg, Germany), anti-Glut4 (sc-53566, Santa Cruz Biotechnology), anti-InsRβ (sc-57342, Santa Cruz Biotechnology), and anti-Vinculin (EPR8185, abcam, Cambridge, UK).

### Fecal microbiota

Stool samples were collected from mice fed either a standard diet with water or TM at baseline (week 0) and week 11 and subjected to shotgun metagenomics sequencing. Microbiome DNA extraction of fecal samples included a course of enzymatic lysis (lysozyme, 100 mg/mL; lysostaphin, 1 mg/mL; mutanolysin, 25 KU/ml, Sigma-Aldrich, 30 min at 37 ºC) and bead beating (FastPrep-24-5G, 1 round of 60 s at 6.0) followed by processing of samples using the QIAsymphony PowerFecal Pro DNA Kit (Qiagen) robotic magnetic bead-based kit. DNA concentrations were assayed using the Qubit dsDNA System, and samples were normalized accordingly to generate high-quality functional libraries using the Nextera XT Library kit (Illumina San Diego, CA, USA), following the manufacturer’s instructions. Library quality control was ensured by profiling and length distribution analysis using the HSD5000 kit in the TapeStation 4200 system (Agilent, Santa Clara, CA, USA). The Libraries were sequenced on a NovaSeq 6000 platform in a 150 paired end reads attaining a minimum of 20 million reads per sample. The configuration generated *.bcl files as primary sequencing output (NovaSeq Control Software v.1.6). The Bcl2fastq v.2.20 program was used to translate the sequencing reads from the bcl to the FASTQ format. This step also removed sequencing adapters.

The Clumpify tool from the BBToolssuite [7] was used to remove optical duplicates. Reads with Phred quality of less score than Q20 and shorter than 50 nucleotides were filtered out using the BBMap program v38.36 [6]. Mice genome presence was filtered using NGLess (v1.0.0) [6, 9], using the built-in *Mus musculus* GCF_000001635.27_GRCm39 genome as a reference. Those reads with alignments with more than 45 bases and 97% similarity to the reference genome were discarded.

Metaphlan v.4.0.6 was used to assign the taxonomy to the reads [4]. The reads of each sample were aligned against the ‘CHOCOPhlAn’ SGB vDec22 database that contains single‐copy genetic markers present in almost all bacteria. From these alignments, the estimated number of reads contributing to a given clade for each identified taxon was obtained computationally.

The MEGAHIT genome assembler (v.1.2.9) [21] was used to perform the assembly of the 'High-Quality Sequences'. Contigs larger than 500 bp were used to predict genes using Prodigal (v2.6.3) [19]. KEGG functional annotation was performed on the predicted genes using the web server GhostKoala [20]. Gene quantification was calculated with SALMON software (v. 1.5.1) [30].

### Statistical analysis

The relative taxonomic abundances of the samples were displayed with collapsed histograms plotted by the 'ggplot2' (v.3.4.0) Library in R version 4.2.3 (R Foundation for Statistical Computing, Vienna, Austria, https://www.R-project.org) [42]. Taxa and gene data were normalized using the rarefaction technique from the 'phyloseq' (v.1.34) R package [27] in order to perform alpha diversity analysis. The effects of the factors on taxonomy and gene data were evaluated with a permutational multivariate analysis of variance (PERMANOVA) with the 'vegan' (v.2.5–7) R package [13] using the Bray–Curtis dissimilarity matrix that was previously calculated considering the relative abundances of taxa and genes in all samples. Additional supporting information related to the analysis of taxa and gene abundance can be found online in the Supporting Information section.

Biochemical and [^18^F]FDG uptake data were log-transformed and the statistical analyses were performed using Prism software (GraphPad Software, Boston, MA).

## Results

### Differential effect in AIP and WT mice following TM diet at libitum

**Fig. 2:**
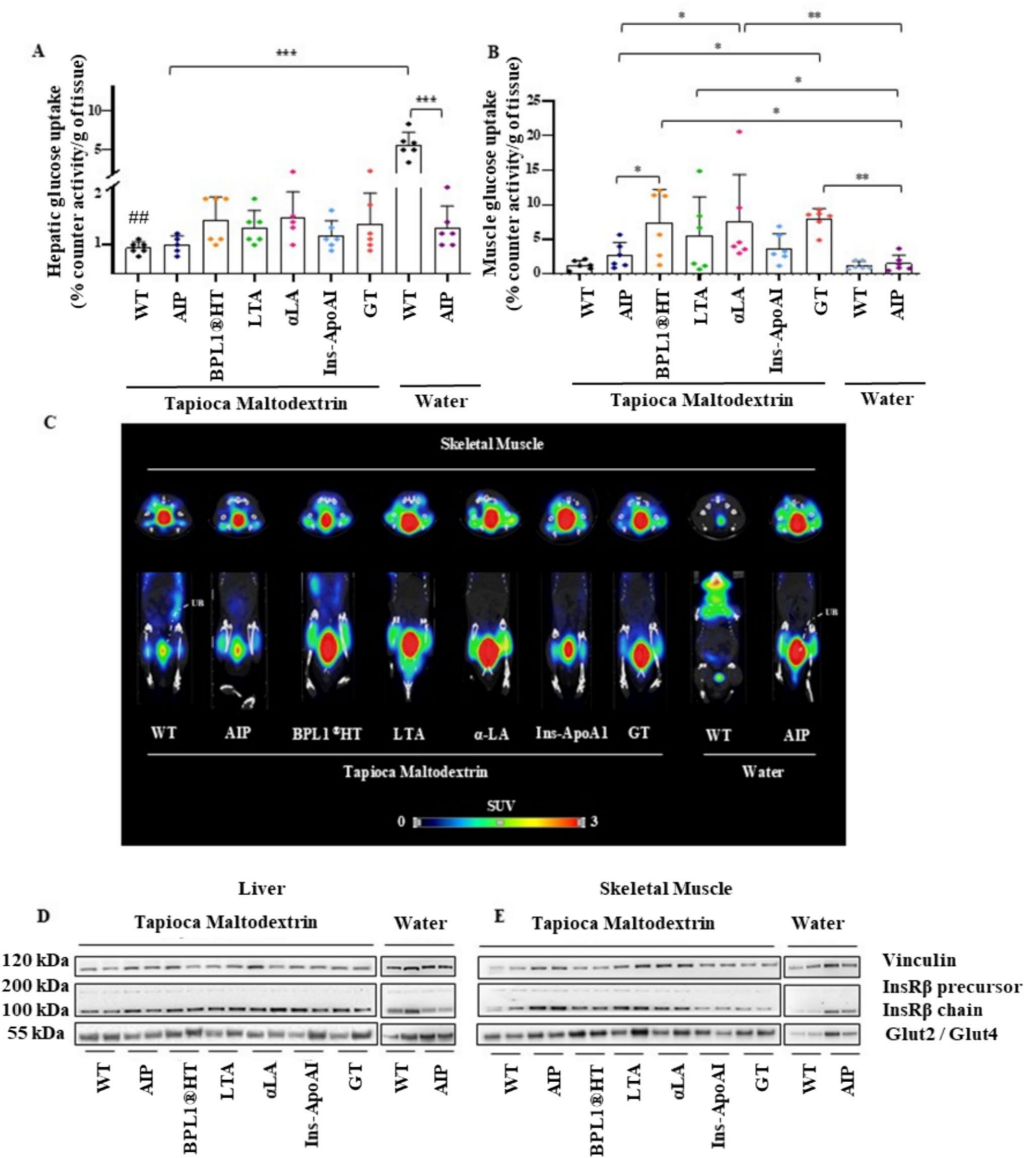
Effects of insulin-sensitizing compounds on [^18^F]FDG uptake and glucose transporters in liver and skeletal muscle. **A**) Ex vivo measurement of [^18^F]FDG radioactivity in murine liver tissue. **B**) Ex vivo quantification of [^18^F]FDG uptake in the skeletal muscles. **C**) Representative PET/CT in vivo images of the skeletal muscle (tibialis anterior): axial view (top); and coronal view (bottom), acquired 1 h after [^18^F]FDG injection in fasted mice. **D**) Western blot analysis of hepatic Glut2 and InsRβ chain. **E**) Western blot analysis of Glut4 and InsRβ chain expression in skeletal muscle. Ex vivo data were normalized to [^18^F]FDG injected volume and tissue weight (g). All mice were fasted 14 h before the sacrifice. Data are shown as mean ± SD. Statistical comparisons were performed using one-way ANOVA followed by Bonferroni post-test, respectively. *, *P* < 0.05; **, *P* < 0.01; ***, *P* < 0.001. ^##^, *P* < 0.01 in WT versus WT-TM

**Fig. 3:**
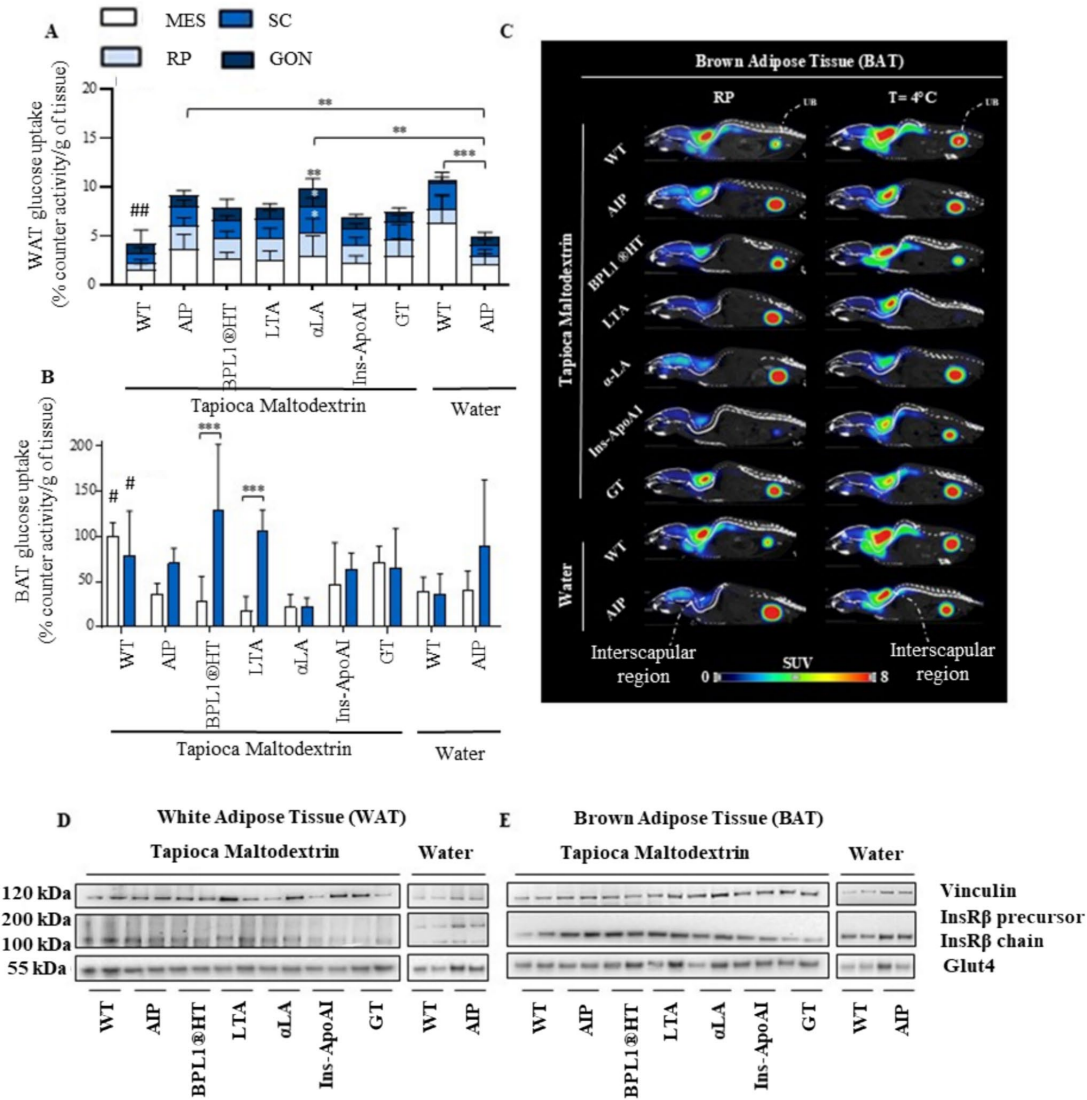
[^18^F]FDG uptake and glucose transporter expression in white (WAT) and brown (BAT) adipose tissues. **A**) *Ex vivo* quantification of [^18^F]FDG uptake in WAT depots (mesenteric (MES), gonadal (GON), retroperitoneal (RP), and subcutaneous(SC)). **B**) *Ex vivo* quantification of [^18^F]FDG uptake in BAT, measured at room temperature (RT, white column) and 4 °C (blue column) in fasted mice. Data were normalized to [^18^F]FDG injected volume and tissue weight (g). **C**) Representative sagittal PET/CT in vivo images showing [^18^F]FDG uptake in interscapular BAT. **D**) Western blot analysis of Glut4 and InsRβ chain expression in WAT (gonadal fat), and E) BAT. Data are shown as mean ± SD. Statistical comparisons were performed using one-way ANOVA followed by Bonferroni post-test, respectively. **, *P* < 0.01; ***, *P* < 0.001. ^#^, *P* < 0.05; ^##^, *P* < 0.01 WT vs WT-TM.

**Fig. 4:**
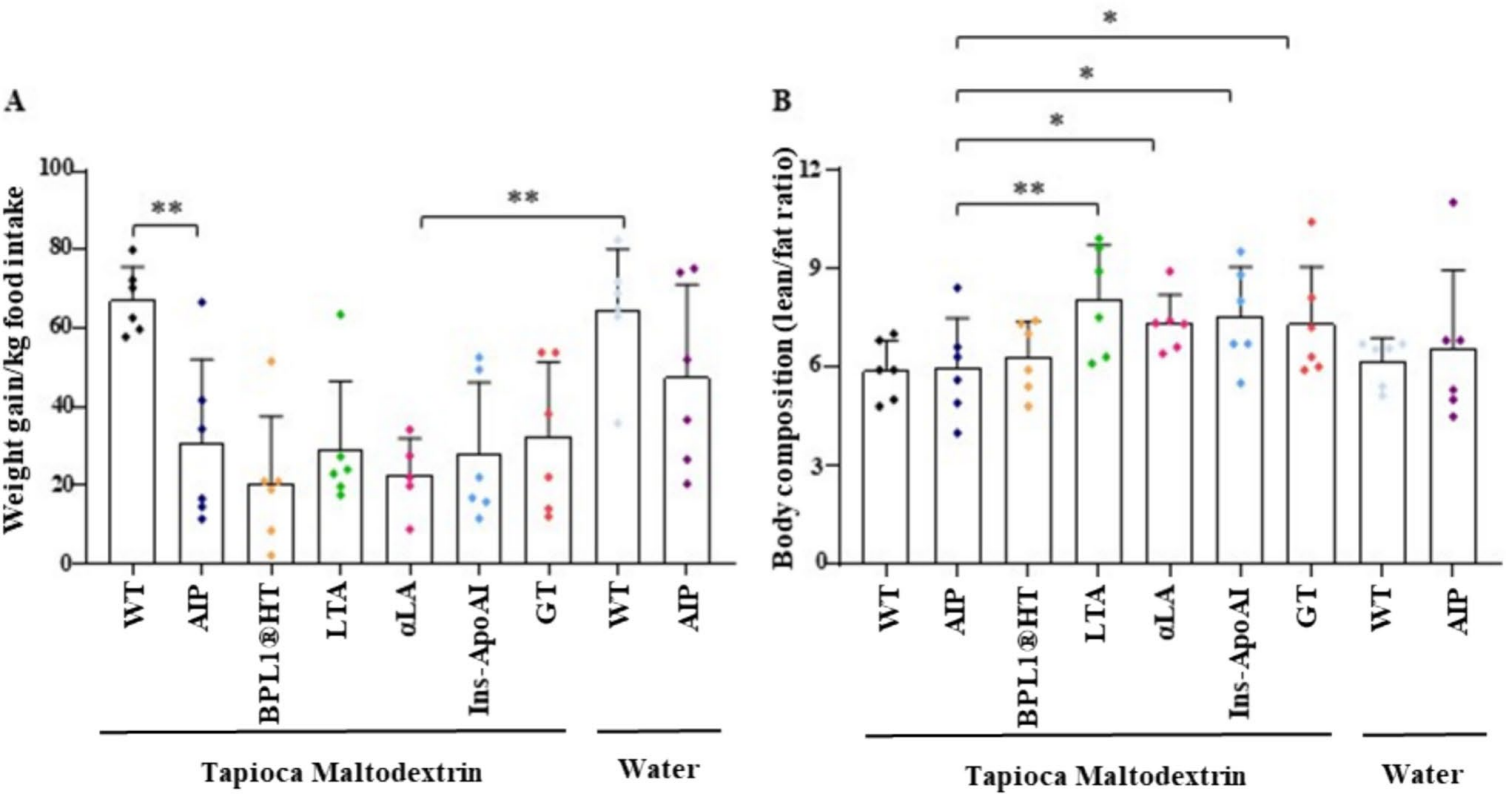
LTA and α-LA diets increase lean-to-fat ratio. **A**) Weekly body weight gain normalized to food intake. Food consumption was recorded three times per week, and weekly intake was calculated as the sum of these measurements. **B**) Body fat percentage and lean mass were measured using EchoMRI-100–700 (Echo Medical Systems, Houston, TX, USA). Data are shown as mean ± SD. Statistical comparisons were performed using one-way ANOVA followed by Bonferroni post-test, respectively. *, *P* < 0.05; **, *P* < 0.01

In animals fed a standard diet and water, AIP mice exhibited elevated insulin levels and impaired GTT compared to WT controls (Fig. 1B, C). Porphyria mice demonstrated reduced [^18^F]FDG uptake in the liver (Fig. 2A) and WAT (Fig. 3A), while uptake in muscle (Fig. 2B, C) and BAT (Fig. 3B, C) was comparable to WT animals. The expression of the insulin receptor (InsR) B chain was decreased in the liver (Fig. 2D), unchanged in WAT (Fig. 3D), but increased in the skeletal muscle (Fig. 2E) and BAT (Fig. 3E) of AIP mice relative to WT controls. Additionally, the lean/fat ratio remained similar between the two strains, although AIP mice tended to show reduced BW gain relative to food intake (Fig. 4A, B).

In WT mice, the carbohydrate-rich diet did not alter serum insulin levels or GTT (Fig. 1B, C). However, [^18^F]FDG uptake was markedly reduced in the liver (Fig. 2A) and WAT (Fig. 3A) while increased uptake in BAT (Fig. 3B). These findings suggest that the TM diet in WT mice may saturate hepatic glycogen storage capacity, leading to increased accumulation in adipose tissues.

In AIP mice, the TM diet significantly increased serum insulin levels compared to AIP mice on a standard diet (Fig. 1B). However, the carbohydrate-rich diet enhanced glucose uptake in WAT (Fig. 3A) while remaining unchanged in the liver (Fig. 2A), muscle (Fig. 2B), and BAT (Fig. 3B, C). These findings highlight distinct tissue-specific glucose uptake responses between WT and AIP mice under a high-carbohydrate diet. Notably, the TM diet did not affect body composition, as assessed by the lean/fat ratio, in either WT or AIP mice (Fig. 4B).

### LTA and α-LA mitigated hyperglycemia, improved glucose tolerance test and enhanced body composition in AIP mice

In AIP mice fed TM diet, supplementation with LTA and *α-*LA fully normalized hyperinsulinemia and glucose AUC values to levels observed in WT animals (Fig. 1B, C). BPL1®HT supplementation demonstrated a trend towards reduced serum insulin levels (Fig. 1B) but retained impaired GTT (Fig. 1C). Liver-targeted therapies, including GT and Ins-ApoAI, provided only partial efficacy in reducing GTT (Fig. 1C).

Weekly food intake was comparable across all groups (90–100 g per week), including those animals drinking water (Fig. 4A). These results indicate that oral supplements and therapies did not influence food consumption. Notably, the lean/fat ratio increased by 33.8% in the LTA-treated group and approximately 20% in the α-LA-treated mice and those receiving liver-targeted therapies, compared to untreated AIP animals on a TM diet (Fig. 4B).

### BPL1®HT and LTA improved muscle glucose uptake and promoted fat disposal in BAT, while the α-LA supplement enhanced glucose uptake in muscle and WAT

In skeletal muscle, [^18^F]FDG uptake was significantly increased in AIP mice supplemented with BPL1®HT, LTA, α-LA and GT compared to AIP (Fig. 2B, C). Moreover, a higher signal of the insulin-sensitive Glut4 transporter was observed in the skeletal muscle of AIP mice supplemented with LTA and the BPL1®HT diet, relative to those fed the TM diet (Fig. 2E).

In the liver, LTA and α-LA supplementation tended to increase InsRβ chain expression, while the BPL1®HT diet, Ins-ApoAI and GT showed trends towards enhancing Glut2 protein expression in AIP livers (Fig. 2D). However, none of the treatments significantly improved [^18^F]FDG uptake in the liver of AIP mice (Fig. 2A).

To assess the role of WAT and BAT under high-carbohydrate diet, we measured [^18^F]FDG uptake in specific fat depots. In AIP mice, higher radiotracer signals in WAT were primarily attributed to the TM diet rather than treatment effects (Fig. 3A). Notably, α-LA increased [^18^F]FDG uptake in gonadal, retroperitoneal, and subcutaneous WAT, but not in mesenteric adipose tissue (Fig. 3A). However, this increase induced by α-LA was independent of InsRβ chain and Glut4 expression (Fig. 3D).

To activate BAT, half of the animals (*n* = 3/group) were exposed to cold for 1 h prior to [^18^F]FDG injection, while the remainder were kept in warm conditions. At room temperature, WT mice fed TM displayed the highest [^18^F]FDG signal (Fig. 3B, C), while cold exposure remarkably increased [^18^F]FDG uptake in the BAT of AIP mice, particularly in those treated with BPL1®HT and LTA (Fig. 3B). Notably, AIP mice fed the BPL1®HT and LTA diets displayed elevated expression of Glut4 in BAT but not in WAT tissues (Fig. 3D, E).

### Taxonomic and functional signatures in fecal microbiome analysis of AIP mice

**Fig. 5:**
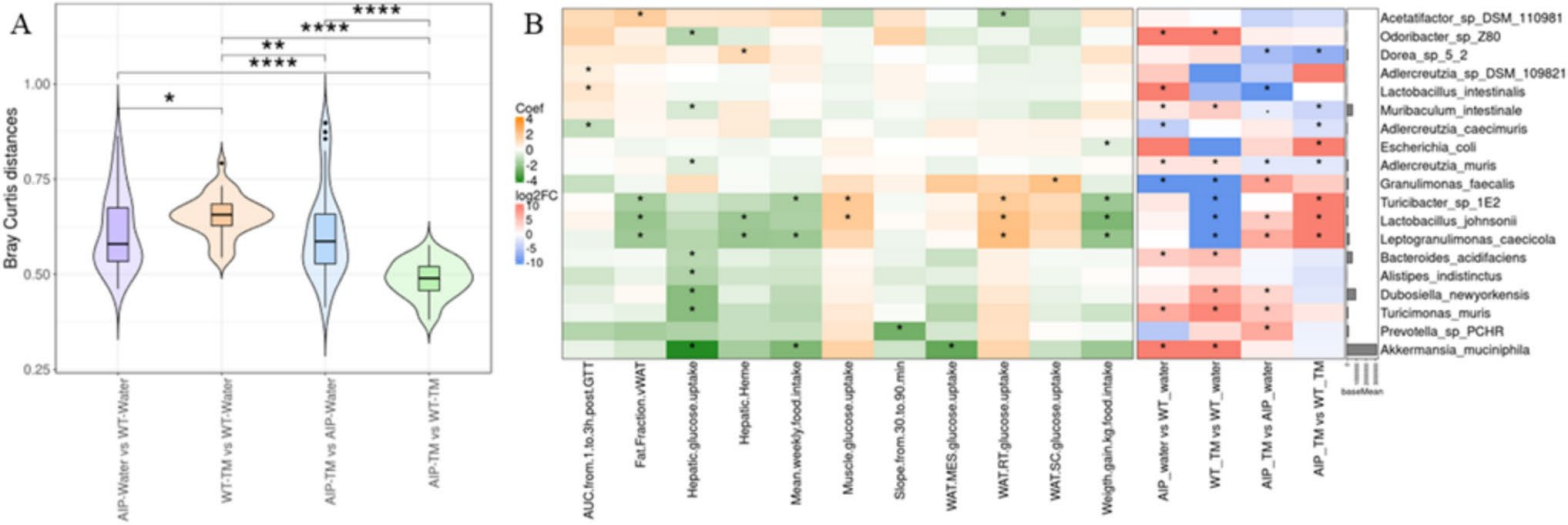
Impact of AIP and tapioca maltodextrin diet on fecal bacterial profiles and correlation between clinical and taxonomic biomarkers in mouse models. (**A**) Bray–Curtis distances of the bacterial taxonomic profile between experimental groups. Statistical significance was tested in R using the Wilcoxon test (stats package). * *p* < 0.05, ** *p* < 0.01, **** *p* < 0.0001. (**B**) Left: Heatmap of correlation coefficients between clinical markers and species-level bacterial abundance, Right: Heatmap of logFC from group comparisons at the species level. Orange indicates positive correlation; green a negative correlation. Significance on the right panel was calculated only for taxa present in at least 50% of samples in one of the compared groups. The accompanying bar plot shows the mean normalized abundance of each taxon. Correlation significance was tested using the Maaslin2 package in R (internal statistical test), while group comparisons were analyzed using the DESeq2 package (Wald test). *, adjusted *p* < 0.05

A total of 406 distinct bacterial species were identified, with the most abundant being *Muribaculaceae* GGB27851_SGB40285 (11.1%), and *Erysipelotrichaceaebacterium* (7.3%). No significant differences in alpha diversity were found between WT and AIP mice on a standard diet with water (Fig. S1A). However, samples from the TM diet clustered distinctly in the upper left quadrant, separating clearly from water-fed samples (Fig. S1B). Bray–Curtis dissimilarity analysis (Fig. 5A) and permutational multivariate analysis of variance (PERMANOVA) confirmed significant microbiome composition differences among groups (R = 0.416, *P* = *0.001*), with the most pronounced alterations in TM diet-fed mice compared to those on a water diet.

Comparing AIP and WT mice on standard diet and water, AIP mice showed a notable enrichment of several species, such as *Bacteroides acidifaciens* and *Adlercreutzia muri* (negatively correlated with hepatic glucose uptake) and *Lactobacillus intestinalis* (correlated with GTT outcomes) (Fig. 5B, **AIP-water vs WT-water**). Conversely, AIP mice showed a significant reduction in *Adlercreutzia caecimuris* (negatively correlated with GTT) and *Granulimonas faecalis* (positively correlated with subcutaneous WAT glucose uptake) (Fig. 5B, **AIP-water vs WT-water**).

Under TM diet, WT mice exhibited an enrichment in several bacterial species negatively correlated with hepatic glucose uptake (including *Bacteroides acidifaciens* and *Adlercreutzia muri,* previously identified in AIP-water mice) and a reduced abundance of species positively correlated with muscle and WAT glucose uptake and negatively associated with hepatic heme levels, such us *Lactobacillus johnsonii *Fig. S2 and Fig. 5B, **WT-TM vs WT-water**).

In AIP mice, TM diet led to an increased abundance of *Prevotella sp PCHR* and *Granulimonas faecalis*, which correlated with altered GTT and subcutaneous WAT glucose uptake, respectively. Conversely, the abundance of species such as *L. intestinalis* or *Dorea sp5.2* decreased, which correlated with altered GTT and reduced hepatic heme levels ( ), respectively (Fig. 5B, **AIP-TM vs AIP-water**). To identify the biomarkers associated with the AIP model under the TM diet, we compared fecal microbiome between AIP-TM and WT-TM groups (Fig. 5B). AIP-TM mice exhibited higher levels of *Escherichia coli* (negatively correlated with weight gain)*,* and *L. johnsonii,* while showing reduced abundance of *Muribaculum intestinale, A. muris,* and *Dorea sp.* 5 2, among others (Fig. 5B, **AIP-TM vs WT-TM**).

**Fig. 6:**
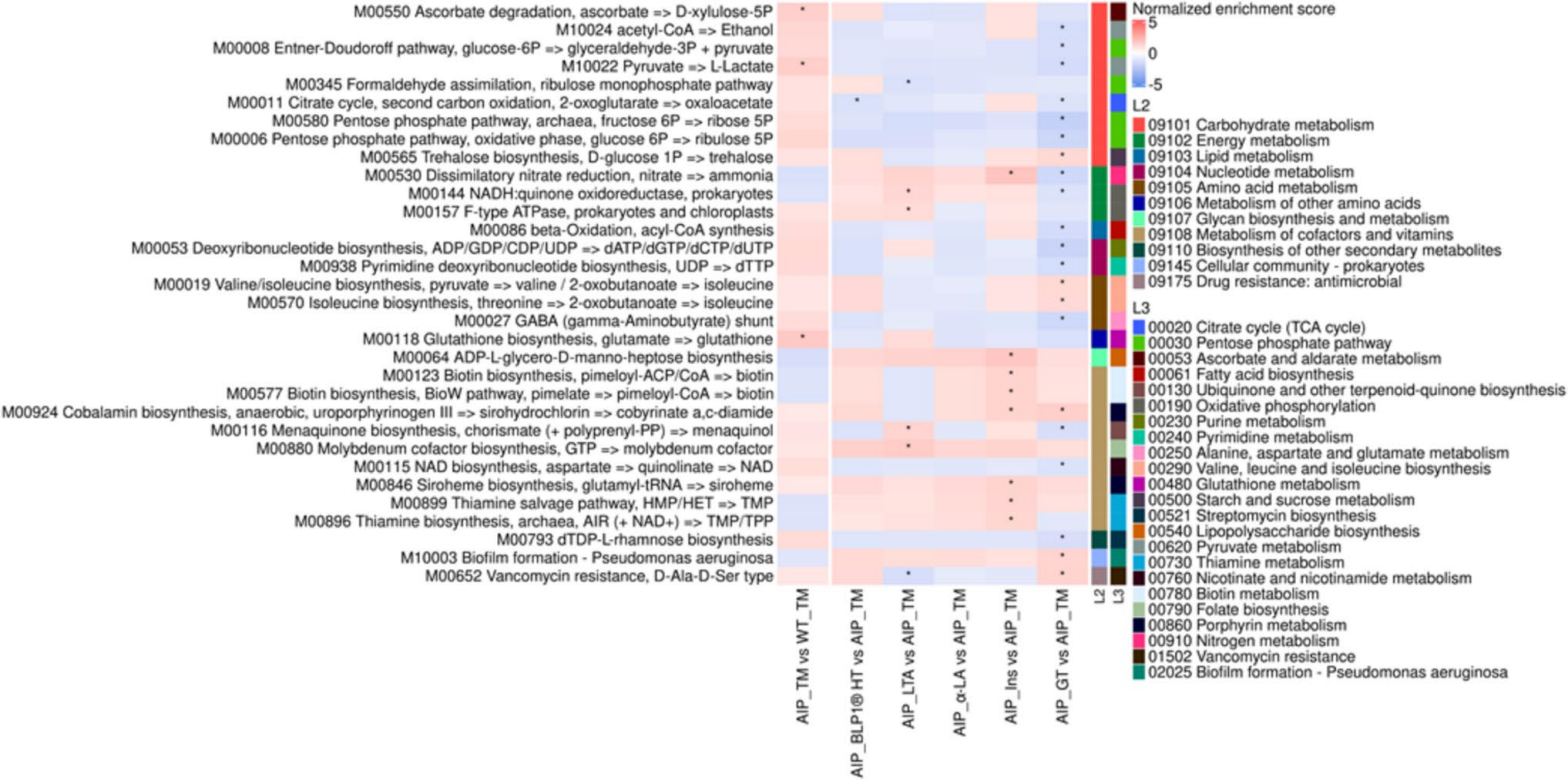
Gene set enrichment analysis of KEGG modules for fecal microbiota in the intervention groups. Heatmap showing the normalized enrichment scores (NES) of KEGG module abundances. Columns represent comparisons between groups. Red shading indicates positive NES values, meaning that the module is enriched in the first group of the comparison. Blue shading indicates negative NES values, meaning that the module is enriched in the second group. The legend ‘L2’ refers to KEGG annotation at the general category level, while ‘L3’ refers to KEGG annotation at the pathway level. Only complete modules are shown. Statistical significance was assessed in R using the fgsea package, with False Discovery Rate (FDR) adjustment. *, adjusted *p* < 0.05

Functional profiling of bacterial communities revealed that the most significant differences were attributed to the TM diet (Fig. 6). In WT mice, pathways related to energy metabolism (e.g., M00176, M00144), and the biosynthesis of cofactors and vitamins (M00064, M00577, M00116) were enriched in the TM group. In AIP mice, the TM diet induced increased abundance of functions related to pyruvate metabolism (M10022), and glutathione synthesis (M00118) compared to WT-TM mice (Fig. 6).

### Impact of dietary intervention on the taxonomic and functional signatures of the fecal microbiome in AIP mice on a TM diet

**Fig. 7:**
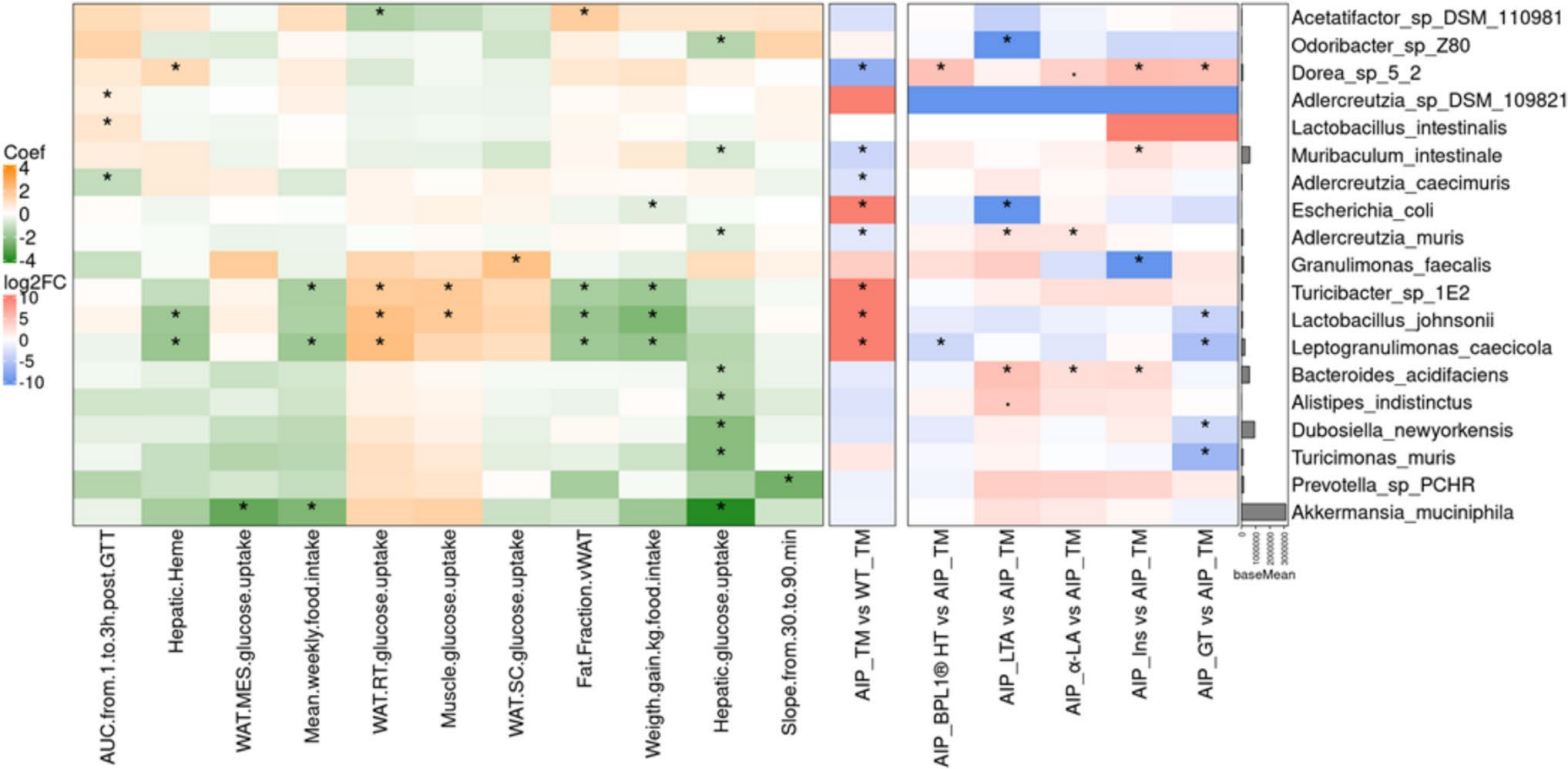
Correlation between clinical and taxonomic biomarkers in the intervention groups. Left: Heatmap of the correlation coefficients between clinical markers and species abundance, Right: Heatmap of the logFC from group comparisons at the species level. Orange shading indicates a positive correlation, while green indicates a negative correlation. In the right panel, significance was calculated for taxa present in at least 50% of the samples in one of the two compared groups. The bar plot shows the mean normalized abundance of each taxon. Statistical significance for correlations was assessed using the Maaslin2 package in R (internal test), and for group comparisons, significance was determined using the DESeq2 package (Wald test). *, adjusted *p* < 0.05

Taxonomic and functional analysis of the fecal microbiome following dietary intervention in AIP mice on a TM diet revealed the highest richness and alpha diversity in liver-targeted therapies, with the GT group showing the great effect, followed by Ins-ApoAI recurrent administration (Fig. 3A). GT treatment increased the abundance of *Dorea* sp. (correlated with hepatic heme)*,* while reducing the abundance of *L. johnsonii and Leptogranulimonas caecicola*, both of which were previously enriched in the AIP fecal microbiome (Fig. 7). Meanwhile, multiple-dose administration of Ins-ApoAI increased the abundance of *Dorea* sp and *M. intestinale*. (Fig. 7), species that were reduced in AIP-TM mice compared to the WT-TM group.

The AIP-TM group supplemented with LTA exhibited the lower diversity, as indicated by Simpson and Shannon index values (Figure S3A). Although PCoA showed no significant sample clustering (Figure S3B), PERMANOVA confirmed significant differences in microbiome composition among the groups (R = 0.375, *p* = *0.001*). Finally, Bray–Curtis distances revealed that the fecal microbiota of the LTA group most closely resembled the WT-TM microbiota (Figure S4).

The administration of LTA and α-LA resulted in an increased abundance of *A. muris* and *B. acidifaciens,* both of which are biomarkers negatively correlated with hepatic glucose uptake. While the α-LA group also showed an increased abundance of the *Dorea* sp. 5 2 (which correlated with hepatic heme), the LTA group showed a significant reduction in *E. coli*, inversely correlated with weight gain relative to food intake (Fig. 7). GSEA analysis indicated that while α-LA and BPL1®HT supplementation resulted in minimal significant changes, the LTA group exhibited enrichment in the oxidative phosphorylation pathway (M00144, M00157) (Fig. 6).

## Discussion

In AIP mice, the addition of the TM diet significantly increased serum insulin levels and worsened glucose tolerance test. Our findings also demonstrate tissue-specific differences in glucose uptake between WT and AIP mice under a high-carbohydrate diet. WT mice showed increased glucose uptake in BAT, whereas AIP mice exhibited elevated uptake in WAT, with no significant changes in liver, skeletal muscle or BAT. At the microbiome level, AIP-TM mice exhibited substantial modifications in the fecal bacterial profile. Notably, their microbiome shifts included significant reduction in bacteria positively correlated with hepatic heme (*Dorea* sp.) and with altered GTT (*L. intestinalis*), while increasing *G. faecalis* (positively correlated with glucose uptake in SC-WAT). At the functional level, AIP mice showed enrichment of genes related to glutathione synthesis and energy metabolism (lactate production), reflecting a metabolic shift aimed at sustaining hepatic ATP production through TCA cycle support, an adaptation aligned with prior findings in AIP models [10, 11, 17].

This study explores oral supplementation with LTA or α-LA in AIP mice on long-term high-carbohydrate diets, revealing their superior ability to mitigate hyperglycemia, enhance glucose tolerance test, and improve body composition (by increasing lean mass relative to fat tissues) compared to rAAV-based GT or a liver-targeted insulin. Hepatocyte transduction with rAAV2/5-*HMBS* restores liver enzyme activity [38], while short-term administration of Ins-ApoAI also alleviates porphyria-associated symptoms by inducing ApoAI-mediated hepatic mitochondrial biogenesis [35]. However, this did not resolve hyperinsulinemia and altered GTT in AIP on a high-carbohydrate diet.

Previous studies have shown that the α-LA insulin-sensitizing agent acts as co-factor of pyruvate and α-ketoglutarate enzymes in the liver, aiding in the replenishment of the hepatic TCA cycle [23, 40]. In *PBGD*-silenced hepatocytes, α-LA enhances glycolysis, ATP production and triglyceride release [23, 25]. In AIP mice, two weeks of α-LA supplementation increased hepatic citrate synthase activity, the rate-limiting enzyme of TCA cycle, and ATP content [23, 25]. Long-term α-LA supplementation also reduced hyperinsulinemia and increased glucose uptake in the skeletal muscle and WAT, as demonstrated by the elevated [^18^F]FDG signal in retroperitoneal, gonadal, and subcutaneous WAT tissues via anatomic-functional PET/CT. Glucose uptake in WAT typically correlates with anabolic activities including lipid storage, glycolysis, and energy production. Similarly, α-LA supplementation in T2DM mice and clinical trials has improved skeletal muscle glucose handling by stimulating Glut4 translocation to cell membranes [33].

Our findings suggest that LTA supplementation, as well as α-LA, enhances energy utilization in the skeletal muscle. LTA is a major cell wall polymer of Gram-positive bacteria, and some studies have reported its fat-reducing properties [2]. Our findings in AIP mice extend this evidence. Intervention with purified LTA demonstrated greater efficacy than BPL1®HT in reducing hyperinsulinemia, normalizing glycemia during the GTT, and improving body composition (evidenced by an increased lean/fat mass ratio despite similar weight gain per kg of food intake). Although LTA is a key effector molecule in BPL1®HT, its concentrations in our assays are likely higher than in BPL1®HT. Moreover, in the postbiotic form, LTA is integrated into the bacterial cell membrane, whereas in the concentrated form it exists in a free state. These variations suggest that different physicochemical states of LTA may result in functional differences. In contrast to LTA, BPL1®HT supplementation was associated with an increased abundance of *Dorea* species, which have been related with the promotion of gut inflammation [32]. Although gastrointestinal manifestations were not observed in AIP mice, gut inflammation may exacerbate such symptoms reported in human AIP, including nausea, vomiting, abdominal distension, and altered bowel habits. Notably, LTA has been shown to enhance gut barrier integrity and support a favorable environment for a healthy intestinal microbiota [41].

Remarkably, LTA administration resulted in a fecal microbiome profile more closely resembling that of WT mice on a TM diet. Many of these changes restored the abundance of bacterial species identified in this study as AIP-TM biomarkers, which were further correlated with enhanced glucose tolerance and insulin sensitivity. Particularly, LTA supplementation led to a reduction in *E. coli* abundance, a species previously reported to be elevated in individuals with diabetes [43].

Further analysis concerning relevant changes in gut bacteria composition is needed in individuals with AIP, preferably through randomized controlled trials. However, dietary interventions were not intended to prevent acute attacks, but rather to improve insulin resistance. None of the oral interventions tested conferred protection against drug-induced acute porphyria attacks, which were prevented only by GT, as previously published [38]. Neither TM diet nor dietary interventions altered the expression of genes regulating the synthesis (*Alas1*) and catabolism of heme (*Ho-1*), or key regulatory genes in energy metabolism (*Pgc1α*)(data not shown).

In conclusion, our findings support the potential of targeted oral interventions, particularly LTA and α-LA supplementation, to improve glucose tolerance, manage insulin resistance, and enhance body composition by increasing lean mass relative to fat tissues. This approach may offer a valuable dietary intervention for the metabolic management of those individuals with AIP for whom high-carbohydrate intake is used to alleviate acute episodes and fasting is contraindicated. However, these results need to be validated in humans through randomized controlled trials.

## Supplementary Information

Below is the link to the electronic supplementary material.

Supplementary file1 (PDF 917 KB)

## Data Availability

All data, except for the raw sequencing information on gut microbiota, are included in the first author's doctoral thesis: Longo, M. Dietary interventions targeting glucose metabolism and hyperinsulinemia: a new translational perspective for the management of Acute Intermittent Porphyria. 2023. Thesis available at: https://air.unimi.it/retrieve/03e22e7c-69db-4242-a3e4-daeb24599129/phd_unimi_R12620.pdf. Raw sequencing data on gut microbiota presented in this study are available upon reasonable request from the corresponding author.

## Abbreviations

AIP: Acute intermittent porphyria
BPL1®HT: Heat-treated *Bifidobacterium animalis* subsp. *lactis* CECT-8145
LTA: Lipoteichoic acid
α-LA: α-Lipoic acid
HMBS: Hydroxymethylbilane synthase
PBG: Porphobilinogen
PBGD: Porphobilinogen deaminase
ALA: δ-Aminolevulinic acid
ALAS: δ-Aminolevulinate synthase
T2DM: Type 2 diabetes
BW: Body weight
WT: Wild-type
TM: Tapioca maltodextrin
rAAV: Adeno-associated vector
GT: Gene therapy
Ins-ApoAI: Insulin-apolipoprotein AI fusion protein
GTT: Glucose tolerance test
MicroPET: Micro-positron emission tomography
[^18^F]FDG: [^18^F]Fluoro-2-deoxy-2-D-glucose
BAT: Brown adipose tissue
WAT: White adipose tissue
GON: Gonadal
RP: Retroperitoneal
MES: Mesenteric
SC: Subcutaneous
InsR: Insulin receptor
PCoA: Principal Coordinates Analysis
GSEA: Gene Set Enrichment Analysis
