# Supplementary material for: Oral lipoteichoic and lipoic acids improve insulin resistance and body composition in porphyria mice on a high-carbohydrate diet
Source: J Physiol Biochem. 2025 Sep 26;81(4):1077–92. doi: 10.1007/s13105-025-01124-4 (PMC12738622; doi:10.1007/s13105-025-01124-4)
Supplement: Supplementary file 1 — Supplementary file1 (PDF 917 KB) [file 13105_2025_1124_MOESM1_ESM.pdf]

SUPPORTING INFORMATION

Additional supporting information relating statistical analysis of taxa and gene abundance.

We performed differential taxa and gene abundance analyses using the DESeq2 R package v.1.30.1[1]. Normalization was based on the 'Relative Log Expression' method. The 'EstimateSizeFactors' function was used to calculate the scaling factors, using the median ratio between taxa/gene abundances and the geometric mean. We used the 'poscounts' method, which deals with taxa/genes that have multiple zeros in most of the samples, as usually occurs in metagenomics. For taxa differential abundance analysis, a taxon was considered differentially abundant if the corrected *p-value* < 0.05 and if it was present in at least 50% of the samples of one of the groups compared.

The MaAslin2 R package (v1.4)[2] was used to study correlations between microbial abundances and clinical variables. A linear model test was performed for each variable, with the variable as a fixed effect.

To analysis the significant differences of the functional profiles between groups, a Gene Set Enrichment Analysis (GSEA) was additionally conducted in the metagenomic dataset using the fgsea (v1.16) R package[3] on KEGG modules. The 'stat' statistic of genes from DESeq2 differential abundance analysis was used to rank the genes to perform the GSEA. Heatmaps were constructed using the ComplexHeatmap R package v.2.11.1[4].

REFERENCES

1. Love MI, Huber W, Anders S (2014) Moderated estimation of fold change and dispersion for RNA-seq data with DESeq2. Genome Biol 15:550.

2. Mallick H, Rahnavard A, McIver LJ, et al. (2021) Multivariable association discovery in population-scale meta-omics studies. PLoS Comput Biol 17:e1009442.

3. Dixon P (2003) VEGAN a package of R functions for community ecology. Journal of Vegetation Science 14:927–930.

4. Gu Z (2022) Complex heatmap visualization. iMeta 1.

5. Oksanen J, Blanchet FG, Friendly M, Kindt R, Legendre P, McGlinn D, Minchin P, O'Hara RB, Simpson G, Solymos P (2016) Vegan: community ecology package. R package version 2.3-5. R Foundation, Vienna, Austria

6. McMurdie PJ, Holmes S (2013) phyloseq: An R Package for Reproducible Interactive Analysis and Graphics of Microbiome Census Data. PLoS One 8:e61217. https://doi.org/10.1371/journal.pone.0061217

7. Wickham H (2006) ggplot: An implementation of the Grammar of Graphics in R. Computer Science

LEGEND TO SUPPLEMENTAL FIGURES

Figure S1. A) Alpha diversity metrics (Richness, Shannon and Simpson) compared between AIP and WT groups under water and TM diet conditions using the Wilcoxon rank-sum test ($p < 0.05$). Richness and Shannon and Simpson indexes were calculated using the 'vegan' (v.2.5-7) R package[5]. Wilcoxon test was performed through the 'stats' (v.3.6.0) R package to find significant differences between groups. B) Principal coordinate analysis (PCoA) plot based on Bray–Curtis distances, illustrating gut microbiome composition across experimental groups. PCoA analysis across was carried out using the Bray-Curtis distance matrix calculated with the 'phyloseq' (v.1.34) R package[6] and represented with the 'ggplot' (v.3.4.0) R package[7]. *P<0.05. WT: wild-type; AIP: Acute Intermittent porphyria; TM: Tapioca maltodextrin

Figure S2. Hepatic heme content was quantified in frozen liver samples using the Heme Assay Kit (Abcam, Cambridge, UK). Data were normalized to total protein content (mg proteins). *, P<0.05; **, P<0.01; ***, P<0.001. WT: wild-type; AIP: Acute Intermittent porphyria; BPL1®HT: heat-treated *Bifidobacterium animalis* subsp. *lactis* CECT 8145; LTA: lipoteichoic acid from BPL1®HT; α-LA: α-lipoic acid; GT: recombinant AAV-*HMBS* mediated gene therapy; Ins-ApoA1: insulin-apolipoprotein A1; TM: Tapioca maltodextrin.

Figure S3. A) Alpha diversity metrics (Richness, Shannon and Simpson) of WT, AIP, and intervention groups under a TM diet, measured using the Wilcoxon rank-sum test (*, p <0.05; **, p <0.01; ***, p <0.001). B) To illustrate fecal microbiome composition dissimilarities based on species and on genes, Principal Coordinates Analysis (PCoA) across the samples was carried out using the Bray-Curtis distance matrix calculated with the 'phyloseq' (v.1.34) R package[6] and represented with the 'ggplot' (v.3.4.0) R package[7]. *, P<0.05; **, P<0.01; ***, P<0.001. WT: wild-type; AIP: Acute Intermittent porphyria; BPL1®HT: heat-treated *Bifidobacterium animalis* subsp. *lactis* CECT 8145; LTA: lipoteichoic acid from BPL1®HT; α-LA: α-lipoic acid; GT: recombinant AAV-*HMBS* mediated gene therapy; Ins-ApoA1: insulin-apolipoprotein A1; TM: Tapioca maltodextrin.

Figure S4. Bray-Curtis distances of the bacterial taxonomic profiles between intervention groups and WT under a TM diet. Statistical significance was assessed using the Wilcoxon test in R (stats package). *, p<0.05. WT: wild-type; AIP: Acute Intermittent porphyria; BPL1®HT: heat-treated *Bifidobacterium animalis* subsp. *lactis* CECT 8145; LTA: lipoteichoic acid from BPL1®HT; α-LA: α-lipoic acid; GT: recombinant AAV-*HMBS* mediated gene therapy; Ins-ApoA1: insulin-apolipoprotein A1; TM: Tapioca maltodextrin.

Figure S1

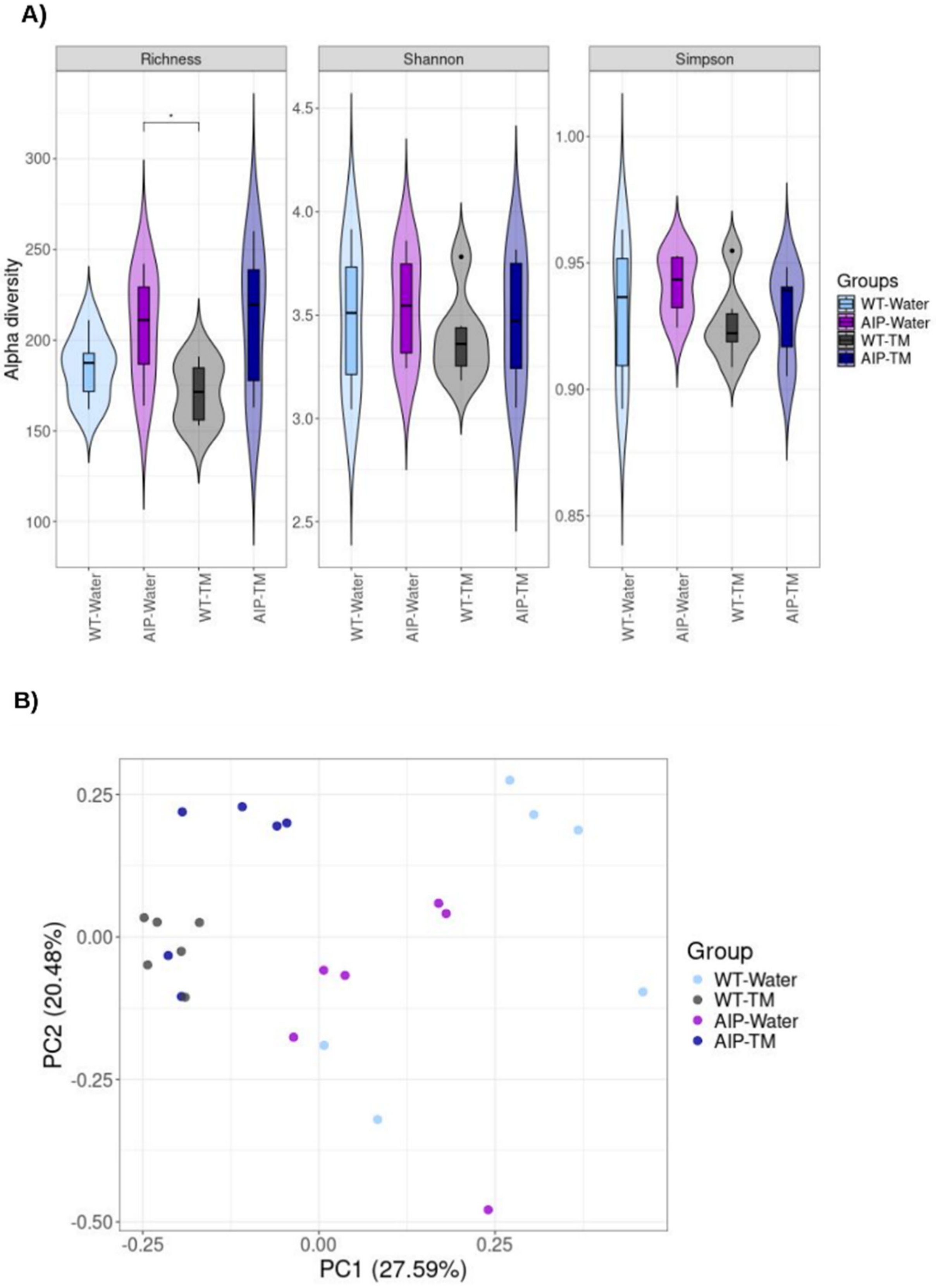
A)
Richness
Shannon
Simpson
Alpha diversity
300
250
200
150
100
4.5
4.0
3.5
3.0
2.5
1.00
0.95
0.90
0.85
WT-Water
AIP-Water
WT-TM
AIP-TM
Groups
WT-Water
AIP-Water
WT-TM
AIP-TM
B)
0.25
0.00
-0.25
-0.50
PC2 (20.48%)
-0.25
0.00
0.25
PC1 (27.59%)
Group
WT-Water
WT-TM
AIP-Water
AIP-TM

Figure S2.

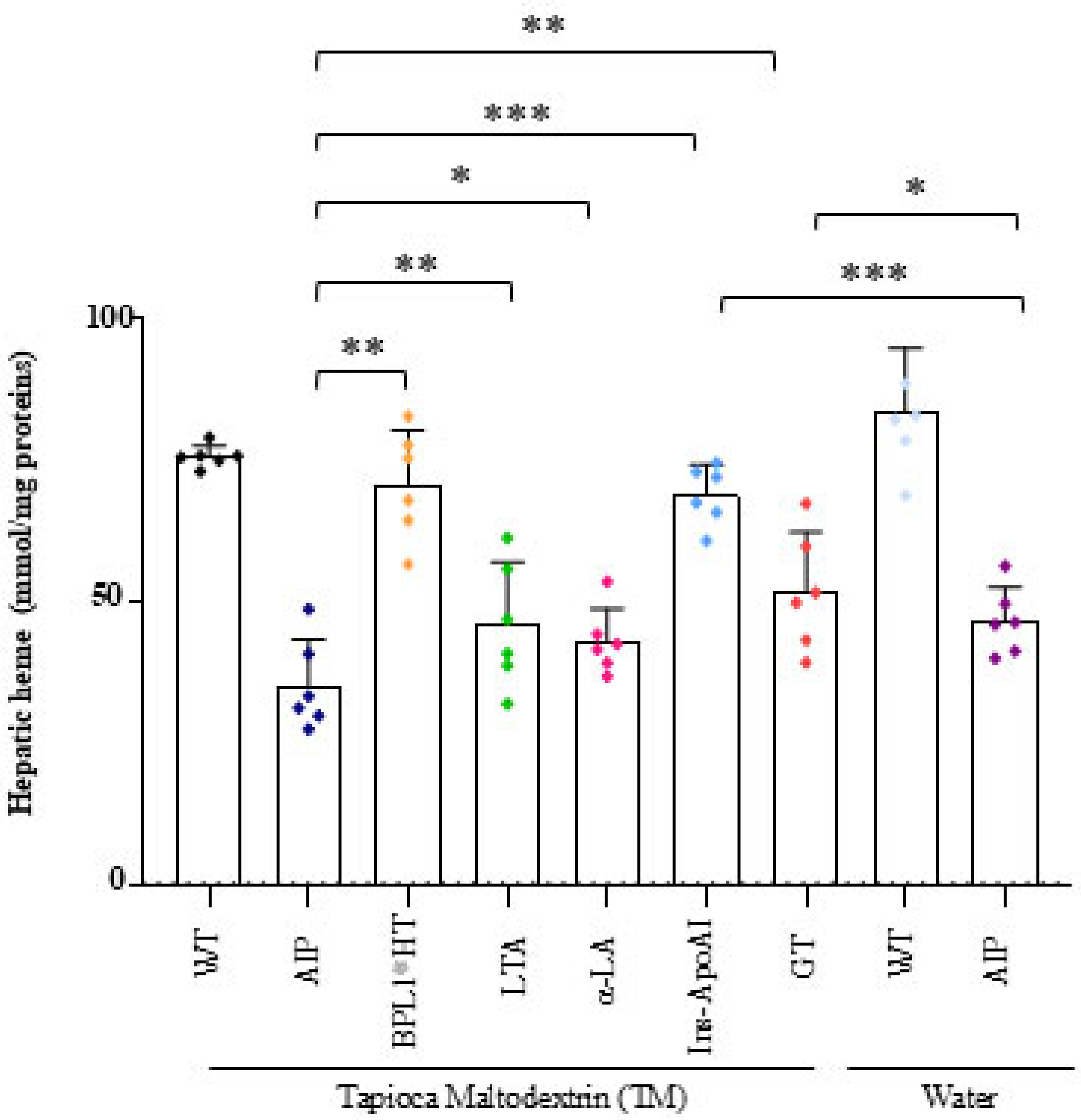

**
***
*
**
**
*
***
100
50
0
Hepatic heme (mmol/mg proteins)
WT
AIP
BPL1®HT
LTA
α-LA
Ins-ApoAI
GT
WT
AIP
Tapioca Maltodextrin (TM)
Water

Figure S3.

A)

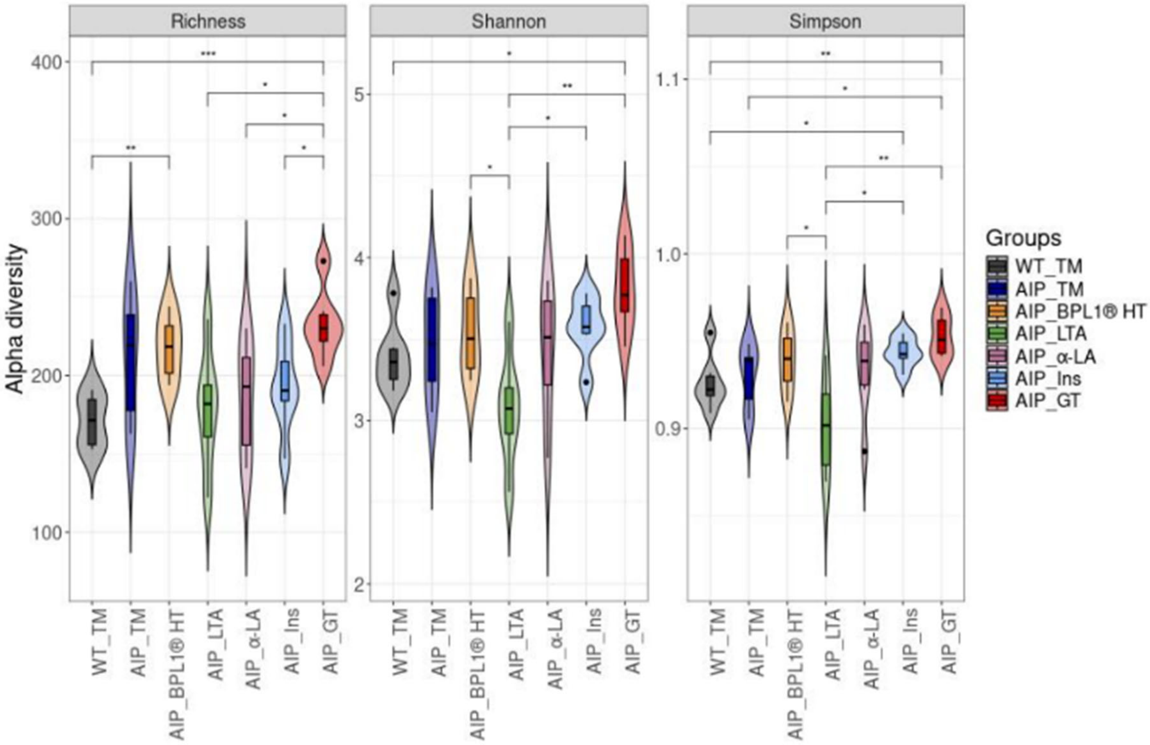
Richness
Shannon
Simpson
Alpha diversity
Groups
WT_TM
AIP_TM
AIP_BPL1® HT
AIP_LTA
AIP_α-LA
AIP_Ins
AIP_GT

B)

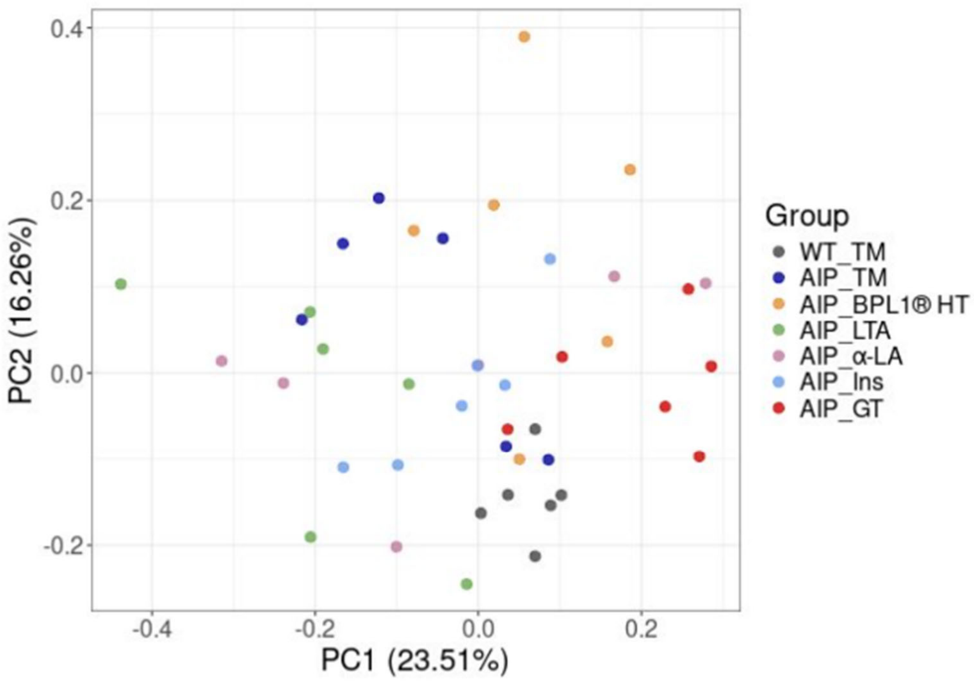
PC1 (23.51%)
PC2 (16.26%)
Group
WT_TM
AIP_TM
AIP_BPL1® HT
AIP_LTA
AIP_α-LA
AIP_Ins
AIP_GT

Figure S4.

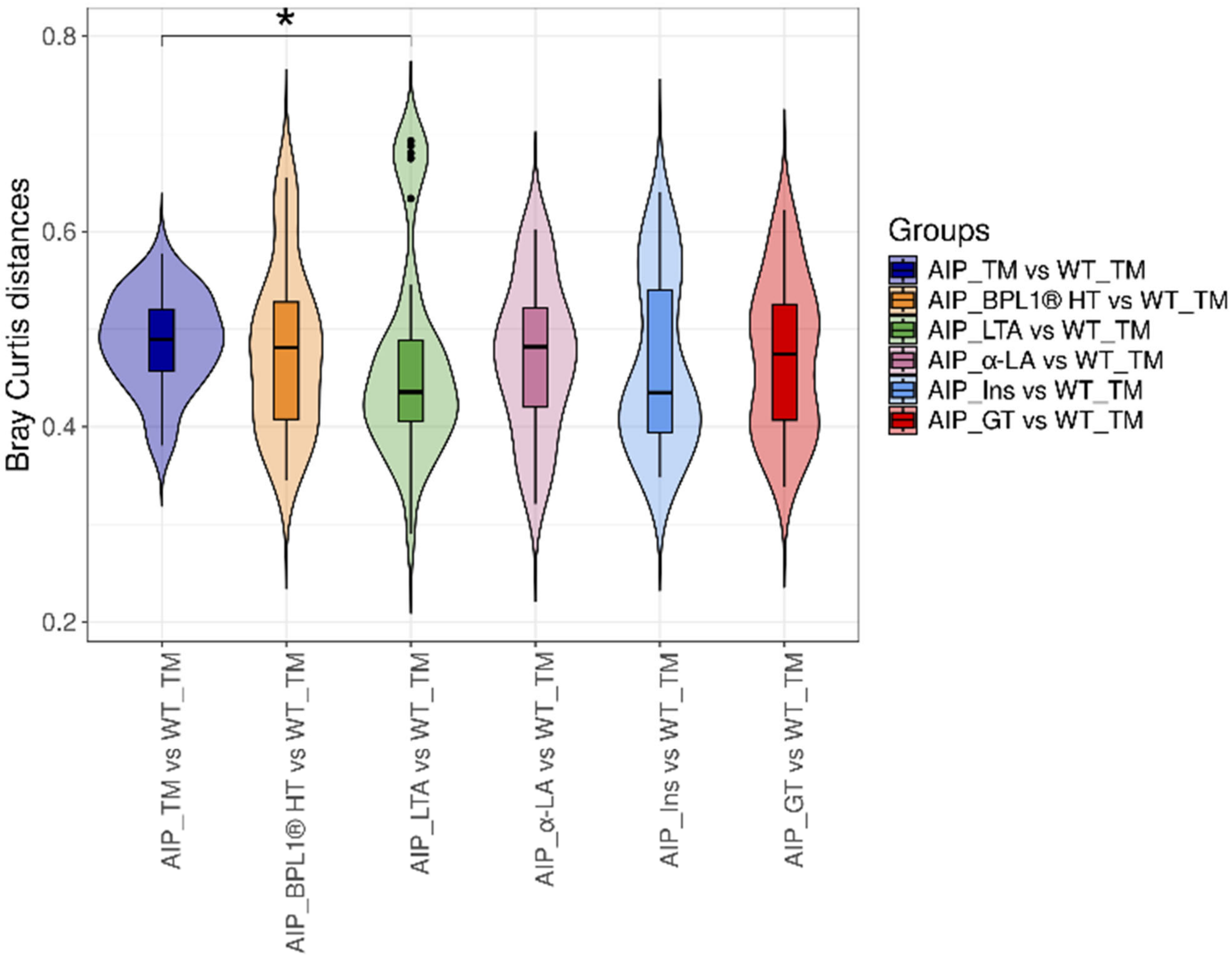
Bray Curtis distances
0.8
0.6
0.4
0.2
*
AIP_TM vs WT_TM
AIP_BPL1® HT vs WT_TM
AIP_LTA vs WT_TM
AIP_α-LA vs WT_TM
AIP_Ins vs WT_TM
AIP_GT vs WT_TM
Groups
AIP_TM vs WT_TM
AIP_BPL1® HT vs WT_TM
AIP_LTA vs WT_TM
AIP_α-LA vs WT_TM
AIP_Ins vs WT_TM
AIP_GT vs WT_TM
